# Temperature-Dependent Conduction and Photoresponse in Few-Layer ReS_2_

**DOI:** 10.1021/acsami.3c12973

**Published:** 2023-10-20

**Authors:** Kimberly Intonti, Enver Faella, Arun Kumar, Loredana Viscardi, Filippo Giubileo, Nadia Martucciello, Hoi Tung Lam, Konstantinos Anastasiou, Monica Craciun, Saverio Russo, Antonio Di Bartolomeo

**Affiliations:** † Department of Physics “E.R. Caianiello”, University of Salerno, Fisciano 84084, Salerno, Italy; ‡ CNR-SPIN, Fisciano 84084, Salerno, Italy; § University of Exeter, Stocker Road 6, Exeter EX4 4QL, Devon, U.K.

**Keywords:** rhenium diselenide, field-effect transistor, Schottky barrier, photoconductivity, temperature, pressure

## Abstract

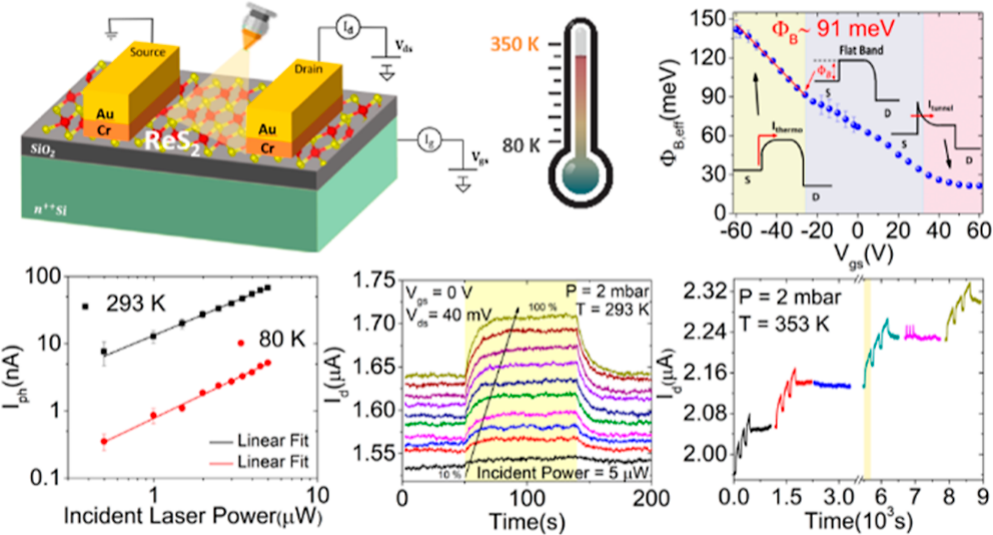

The electrical behavior and the photoresponse of rhenium disulfide field-effect transistors (FETs) have been widely studied; however, only a few works have investigated the photocurrent as a function of temperature. In this paper, we perform the electrical characterization of few-layer ReS_2_-based FETs with Cr–Au contacts over a wide temperature range. We exploit the temperature-dependent transfer and output characteristics to estimate the effective Schottky barrier at the Cr–Au/ReS_2_ interface and to investigate the temperature behavior of parameters, such as the threshold voltage, carrier concentration, mobility, and subthreshold swing. Through time-resolved photocurrent measurements, we show that the photocurrent increases with temperature and exhibits a linear dependence on the incident light power at both low and room temperatures and a longer rise/decay time at higher temperatures. We surmise that the photocurrent is affected by the photobolometric effect and light-induced desorption of adsorbates which are facilitated by the high temperature and the low pressure.

## Introduction

Highly efficient and environmentally friendly photoelectric technologies have received a lot of attention in recent years due to the advancement of light–electricity conversion in nanoscale materials. In particular, two-dimensional materials, like graphene,^1−3^ black phosphorus (BP),^4,5^ and transition-metal dichalcogenides (TMDs),^6−8^ have been widely investigated for optoelectronic applications. Most group-VI TMDs, such as molybdenum disulfide/diselenide and tungsten disulfide/diselenide, exhibit a transition from an indirect to a direct band gap, while their thickness is reduced to a monolayer, which make them suitable to be used as channel materials in field-effect transistors and photodetectors.^9−12^ For instance, molybdenum disulfide (MoS_2_) exhibits a high mobility, a high *I*_on_/*I*_off_ ratio, as well as high photoresponsivity and optical memory performance.^13,14^

Rhenium disulfide (ReS_2_), together with rhenium diselenide^15^ (ReSe_2_), is one of the most recently discovered materials belonging to group-VII TMDs and exhibiting a distorted 1T highly anisotropic in-plane structure. It differs from group VI TMDs because it maintains a direct band gap of ∼1.5 eV and an almost invariant band structure irrespective of the thickness.^16^ Indeed, in its bulk form, it behaves as electronically and vibrationally decoupled monolayers because of the weak interlayer coupling and the lack of interplay registry.^17^ The use of a variable number of ReS_2_ layers can be promising for several applications, such as photodetection.

Zhang et al. fabricated top-gate field-effect transistors via the encapsulation of ReS_2_ nanosheets in Al_2_O_3_. They obtained a strong dependence of the photocurrent, defined as *I_ph_ = I_light_ – I_dark_* on the laser power, attributed to the photogeneration mechanism and a photoresponsivity of 16.14 A/W at 25 nW laser power.^18^ A higher photoresponsivity of 10^3^ A/W was obtained by Liu et al., who investigated the optical properties of 3 nm thin ReS_2_ under a green semiconductor laser of 2.4 eV. They showed that the photocurrent follows a power law as a function of the laser power *I_ph_ ∼ P*^*γ*^, where γ is 0.3. This sublinear dependency was attributed to complex carrier generation, trapping, and recombination processes.^19^

However, both Zhang et al.^18^ and Liu et al.^19^ examined devices fabricated with few-layer ReS_2_, which makes it difficult to absorb sufficient light, as they could not have a real control on the thickness of their mechanically exfoliated ReS_2_ flakes. Shim et al. reported a simple top-down approach for fabricating a photodetector with a controlled thickness above 30 nm. They performed an oxygen (O_2_) plasma treatment to achieve a high *I_on_/I_off_* current ratio, a high mobility, and a photoresponsivity of 10^7^ A/W. This high photoresponsivity was ascribed to the direct band gap of ReS_2_ and the high absorbance of the thick film as well as to the prolonged lifetime of carriers trapped in O_2_ plasma-induced defects.^20^

The above-mentioned investigations of the photoresponse of ReS_2_ were carried out at room temperature. However, there has been increasing interest in the temperature-dependent properties of 2D materials.^21,22^ Actually, the development of photoelectric devices also must deal with their suitability to extreme environmental conditions. Although there has been enough investigation at room temperature, the physical mechanisms affecting the photoresponse at extreme temperatures, especially at low temperatures, are not yet enough understood. In reality, some research works indicate that low temperatures lead to a poor performance because of carrier freezing and low absorption, while other works report that low temperatures improve the photoelectric characteristics.^23^

Pradhan et al. examined the temperature dependence of the electrical properties of ReS_2_, from 300 to 2 K. They determined that the field-effect mobility increases up to 350 cm^2^/(V s) as the temperature is decreased to 100 K because the carrier scattering rate from phonons decreases as the temperature is lowered. Below 100 K, impurity scattering, carrier localization, or the suppression of thermionic emission of carriers across the Schottky barrier cause the mobility to saturate. The threshold voltage increases as well because of the charge localization at the interface with the substrate.^24^

An increase in mobility when the temperature is lowered to 77 K was also reported by Corbet et al. for dual-gated ReS_2_ field-effect transistors.^25^ Similarly, Zhang et al.^18^ showed that the mobility decreases above 120 K because of the electron–phonon scattering, according to the relation μ ∼ *T^–γ^* with γ = 2.6. They also modeled the temperature-dependent conductance G with the equation , obtaining the thermal activation energy of charge carriers to transit into the conduction band. However, they did not examine if the photoresponse of the material could be sensitive to the operating temperature: there is a gap in the literature about the thermally dependent photoresponse of 2D ReS_2_.

In this paper, we first study the electrical properties of ReS_2_ devices in terms of conductance, threshold voltage, carrier concentration, mobility, and subthreshold swing in the temperature range from 180 to 350 K. Using the current at fixed gate voltages, derived from the transfer curves at different temperatures, in an Arrhenius model, we extract the Schottky barrier at the Cr–Au/ReS_2_ interface as a function of the gate voltage. We estimate an effective Schottky barrier lower than that expected based on the work function difference, confirming the occurrence of the Fermi level pinning. We also evaluate the body factor and interface trap density. After that, we investigate the time-resolved photoresponse at 80, 293, and 353 K using light pulses of different intensities and durations. We find a linear dependence of the photocurrent on the light power and a time response depending on the temperature. We ascribe the observed slower rise/decay time at high temperatures to the photobolometric effect and light-induced desorption of adsorbates.

## Results and Discussion

**Figure 1:**
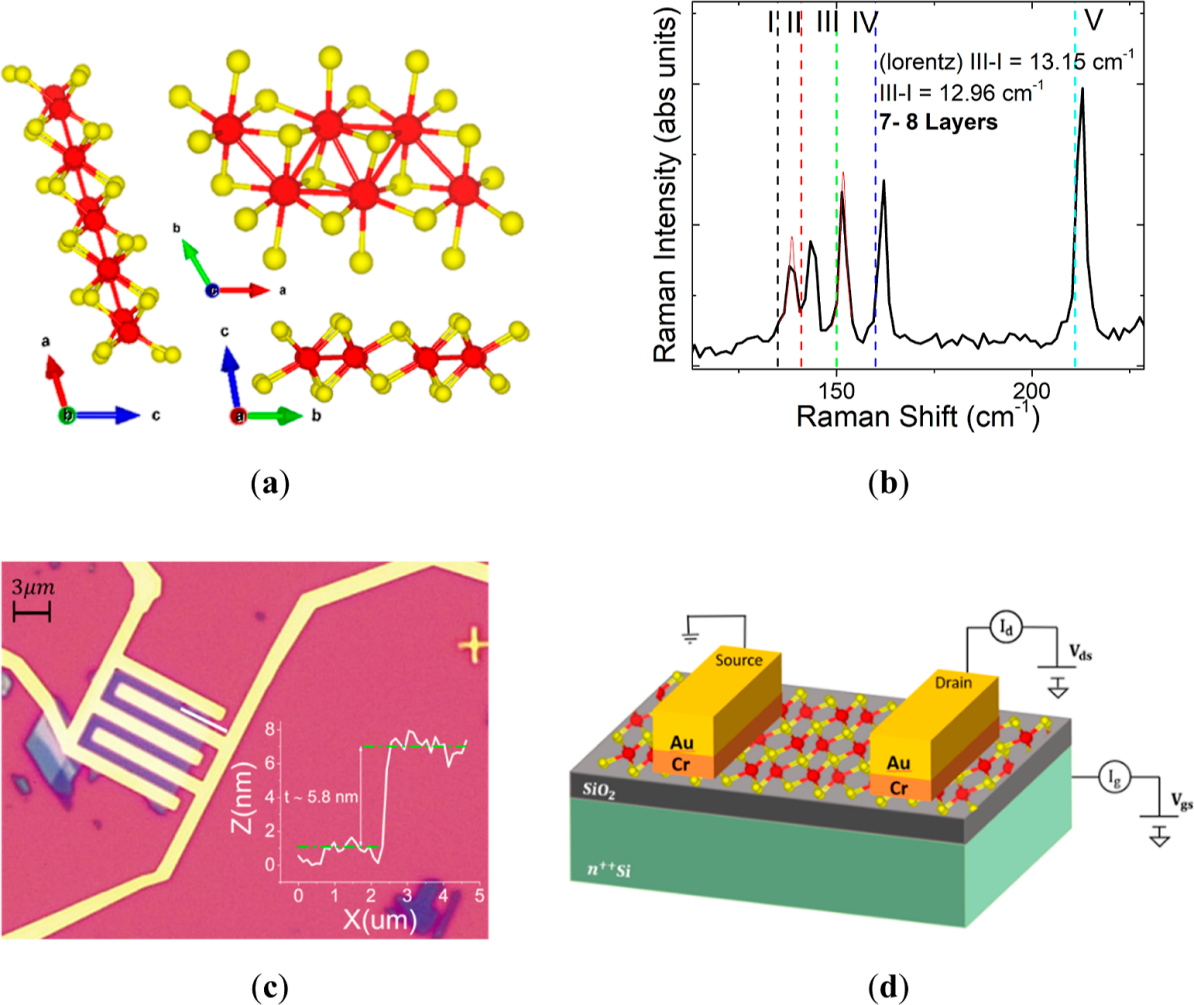
(a) ReS_2_ crystal structure along the *a*, *b*, and *c* axes. The Re–Re bonds, due to the extra valence electron of Re, induce a Re–Re chain along the crystal. (b) Raman spectrum of ReS_2_ restricted between 100 and 250 cm^–1^. (c) Optical image of the device, which shows the interdigitated layout of the metal contacts. In the inset, the AFM profile acquired along the white trace is shown in the optical image. (d) Schematic of the device made of a ReS_2_ flake transferred onto a SiO_2_–Si substrate and in contact with Cr–Au leads. Gate and drain voltages are applied to perform electrical measurements.

Figure 1a reports the atomic structure of ReS_2_, which exhibits a distorted 1T structure. Studies of the bulk form reveal that, due to extra valence electrons of rhenium atoms, ReS_2_ exhibits both metal–chalcogen and metal–metal bonds (in plane Re–Re chain).

Ultrathin ReS_2_ flakes were obtained by mechanical exfoliation from bulk ReS_2_ single crystals. By using adhesive tape, the flakes were transferred onto a highly doped n-type (resistivity: 0.005 Ωcm) silicon substrate, covered by a 290 nm thick SiO_2_ layer, which acts as a global back gate. The Raman spectrum of ReS_2_ (Figure 1b), in the (100–250) cm^–1^ range, where the strongest modes occur, indicated a flake of 7–8 layers. The flake thickness can be extracted from the energetic difference among some high-energy modes of ReS_2_. In detail, the difference between the peak positions of the I and III modes decreases with increasing number of layers, giving a reliable method to estimate this parameter.^26,27^ Raman spectroscopy was performed with a fixed polarization angle. It is noted that the peak intensity ratios are sensitive to the direction of the polarization and are modified even if the crystal is rotated with the same laser and collection polarizations.^24^ This demonstrates the anisotropy of the vibrational properties of ReS_2_.

Back-gated field-effect transistor (FET) devices were fabricated by depositing Cr–Au (5–100 nm) contact leads by thermal evaporation. The electrical transport also depends on the direction; the electrical conductivity along the *b*-axis is several times higher than that on the perpendicular axis. To perform anisotropy transport measurements, many electrode pairs are necessary, which are disposed at different angles. In this case, the two metallic contacts are randomly deposited; therefore, it is not possible to select a single crystalline direction and appreciate the anisotropic electrical properties of the material. We adopted an interdigitated layout, shown in the optical image in Figure 1c, which yields a total length L = 0.89 μm and width W = 42 μm. In the inset, the AFM profile confirms that the flake is 8-layers thick, since the single layer has a thickness of around 0.7 nm.^26^ The schematic of the device is shown in Figure 1d, along with the circuit diagram used to apply the Si/SiO_2_ back gate and the source–drain biases, *V*_gs_ and *V*_ds_, respectively, to the 2D semiconducting channel.

**Figure 2:**
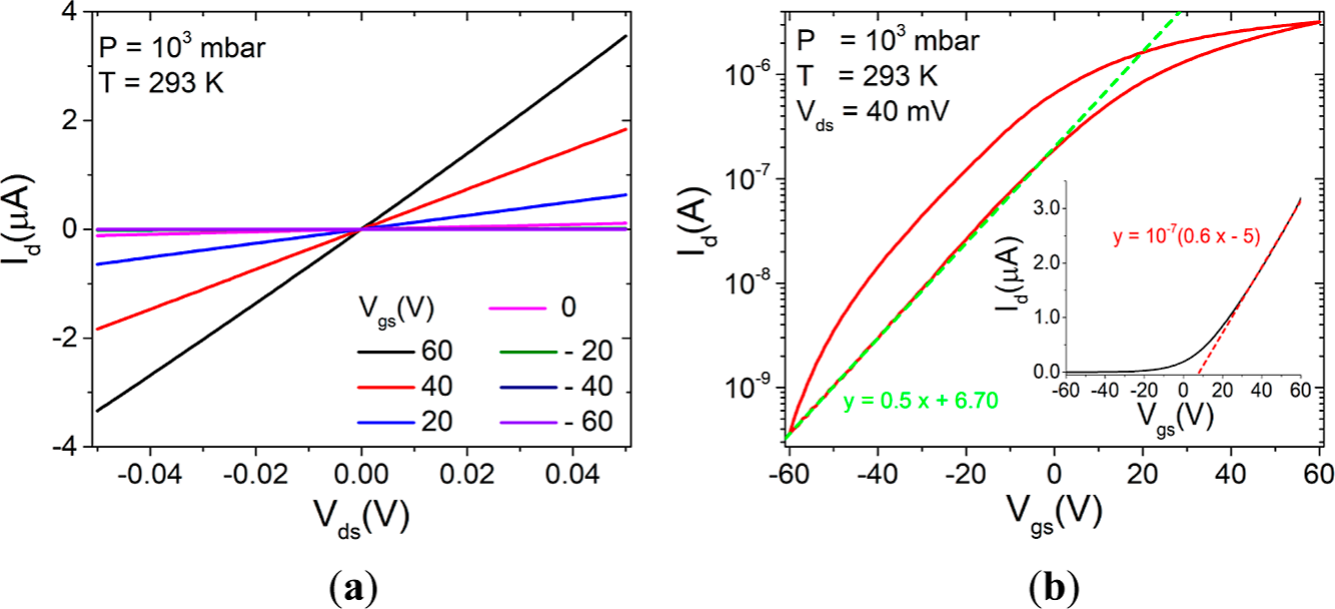
(a) Output curves measured at ambient pressure, by sampling *V*_gs_ in the (−60, 60) V range with steps of 10 V. (b) Transfer curve on a semilog scale and on a linear scale (inset).

Electrical measurements were initially performed in the dark at room temperature and ambient pressure. Figure 2a reports the output curves (*I*_d_–*V*_ds_, where *I*_d_ is the drain current) of the device on a linear scale. They exhibit a symmetrical behavior for positive and negative *V*_ds_ and a linear shape, which indicate negligible Schottky barriers at the Cr–Au/ReS_2_ interface.^19,28^ However, because Schottky interfaces on highly doped semiconductors or low Schottky barriers can originate Ohmic current–voltage curves, the linear behavior is not conclusive about the nature of contacts, and deeper investigations are required.^29^ Additionally, the growing channel current for increasing gate voltage confirms an n-type conduction for the sample.^19,24^ The n-doping of ReS_2_ and for most TMDs is generally attributed to chalcogen vacancies,^30^ which are the most common defects in intrinsic ReS_2_^31^ and, more generally, in mechanically exfoliated TMD flakes.

To get more insights into the behavior of the device, the transfer characteristic was measured, at a fixed *V*_ds_ of 0.04 V. The quality of the switching properties can be evaluated from the semilog *I*_d_–*V*_gs_ plot reported in Figure 2b. The device exhibits not only an *I*_on_/*I*_off_ ratio of about 10^4^ but also a high subthreshold swing of 21.4 V/decade, obtained by the following formula
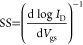
1

Such a high SS might indicate a high density of defects at the interface with SiO_2_, which are also the main cause of the hysteresis. The quality of the interface with the gate dielectric can be improved through the introduction of an ultrathin BN dielectric, which results in higher electrical performances of the device.^32^ The field-effect mobility of ∼3 cm^2^ V^–1^ s^–1^ was extracted from the linear part of the transfer curve on the linear scale, shown in the inset of Figure 2b, according to the formula
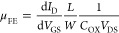
2where *C*_ox_ = 1.15 × 10^–8^ F cm^–2^ is the gate dielectric capacitance. The result is consistent with other works on similar devices^18,19^ and is typical of several TMDs.^13,33,34^ Since high biases were applied, the current through the oxide was also monitored to make sure that no gate leakage was affecting the measurements.

To understand the mechanisms that limit the mobility in ReS_2_, we examined the temperature dependence of the electrical properties in the range 180–350 K. This range was also chosen since it is suitable to carry out a transistor analysis based on the Arrhenius model. Actually, the Arrhenius method is not reliable at low temperatures since the thermionic component results in a smaller current than the usual leakage for any considerable Schottky barrier height.^29^ All the following measurements were performed in vacuum, at 2 mbar, to avoid freezing the chamber during the cooling phase.

**Figure 3:**
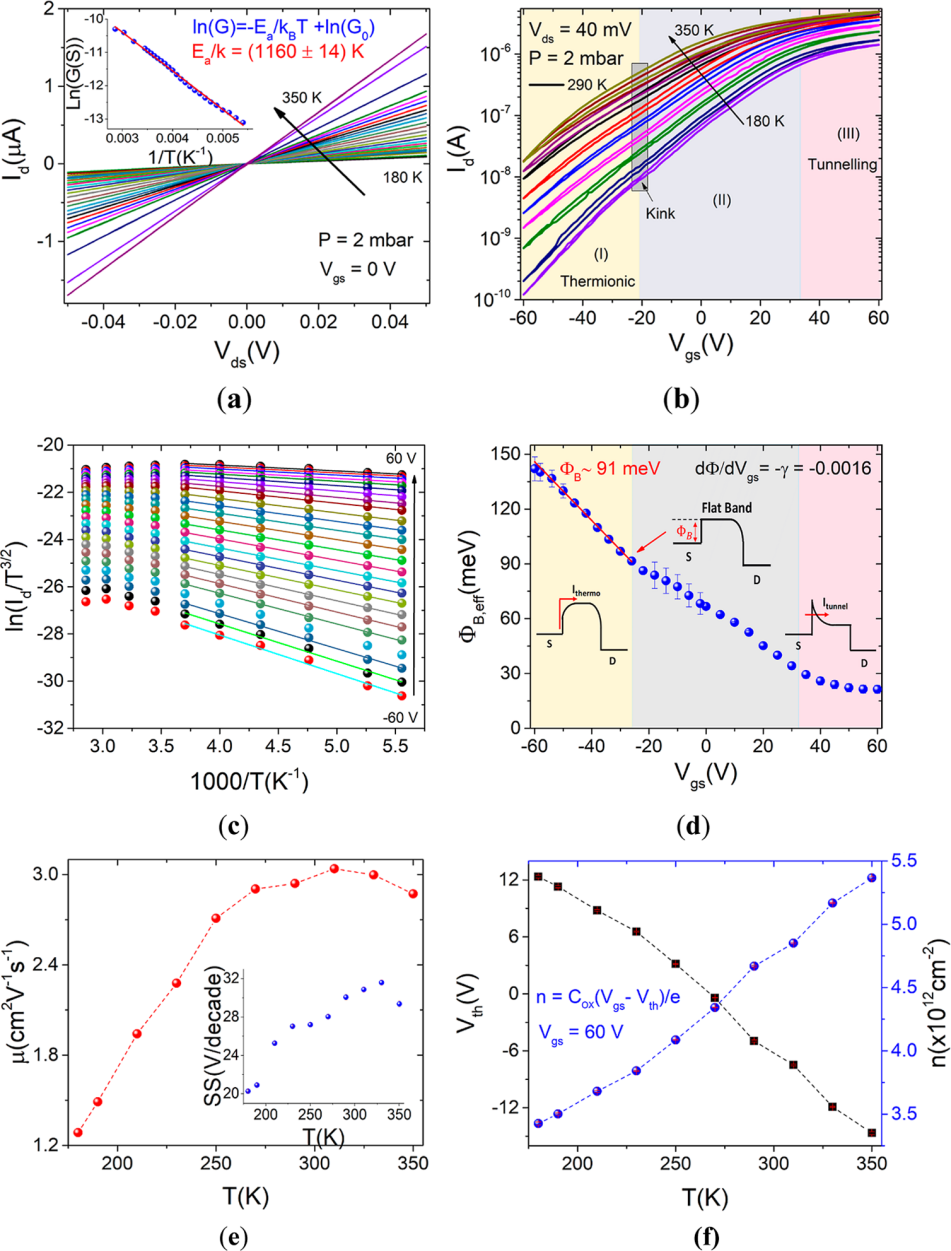
At 2 mbar: (a) Current–voltage (*I*_d_–*V*_ds_) curves obtained at different temperatures, from 180 to 350 K. The inset shows the conductance (*G*) vs 1/*T* plot and is used to extract the activation energy at *V*_gs_ = 0 V. (b) Transfer curves at different temperatures on a semilog scale. The three colored regions qualitatively demark the thermionic (I), mixed (II), and tunneling (III) dominated operation modes. (c) Arrhenius plot. (d) Effective Schottky barrier as a function of the gate voltage, with the three-operation modes shown as colored zones. (e) Mobility and subthreshold swing as a function of temperature. (f) Threshold voltage (black squares) and carrier concentration (blue spheres) as a function of temperature.

Figure 3a reports the *I*_d_–*V*_ds_ curves at zero-gate voltage and for *V*_ds_ ranging from −0.05 to 0.05 V. A linear behavior is exhibited at every temperature. The conductance shows semiconducting behavior as it increases with increasing temperature. The variation of the channel conductance G with temperature can be fitted by the Arrhenius equation , as shown in the inset of Figure 3a. The thermal activation energy of the majority carriers is *E*_a_ = (100 ± 2) meV, revealing the presence of energy levels close to the conduction band that would cause Fermi level pinning and low Schottky barrier.^35^

More insights are provided by the transfer curves at different temperatures measured by forward and reverse sweeping of the gate voltage in the (−60, 60) V range at a fixed *V*_ds_ = 0.04 V. In the plot of the *I*_d_–*V*_gs_ curves of Figure 3b, the three different operation modes that Schottky-barrier MOSFETs usually pass through for increasing *V*_gs_ can be qualitatively identified. For a gate voltage up to about −20 V, carriers are injected in the channel through thermionic emission (TE) from the source (*I*). The lower the gate bias, the deeper the device operates in the off state, and the higher the change in the current as a function of temperature. The conduction band maximum in the channel is located energetically above the Schottky barrier and moves to lower energies for higher gate voltages. As long as the conduction band maximum is above the Schottky barrier, the Schottky barrier does not affect the transistor current as the electron flow is limited by the band profile in the channel. When the conduction band maximum aligns with the Schottky barrier level, the flat band condition is achieved. By further increasing the gate voltage, the conduction band maximum goes below the Schottky barrier and thermal-assisted tunneling (TT) is enabled. Then, the channel current is the result of both TE and TT (II). This transition is identified by a kink in the transfer curves at the gate voltage corresponding to the flat-band voltage. The higher the tunneling probability through the Schottky barrier, the less pronounced is the kink. At higher gate voltages, the conduction band in the channel bends further, making the potential barrier thinner and thinner, thus enhancing the transmission probability through it. When the channel conduction band is slightly above the source Fermi level (threshold condition), the transistor turns on. This manifests as a change in the current from exponential to quadratic/linear behavior (apparent saturation of the drain current at high gate voltages on the semilogarithmic *I*_d_–*V*_gs_ plot in Figure 3b). Above the threshold region, the tunneling mechanism is dominant (III).

The flat-band voltage and the Schottky barrier height affected by the pinning of the Fermi level can be obtained by the Arrhenius analysis.^36^ As already mentioned, in the off state, the current transport at the reverse-biased source junction of a 2D FET is dominated by thermionic emission. In this regime, the current is defined as
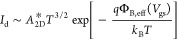
3

To describe the three operating modes, it is convenient to introduce *q*Φ_B,eff_(*V*_gs_), which is the effective barrier height that depends on the shape and the width of the potential barrier seen by the electrons at the source edge, as determined by the conduction band profile at a certain gate voltage. *A*_2D_* is the modified Richardson constant and *T* is the temperature. In the TE regime, the current exponentially depends on the temperature and gate voltage. At the flat-band voltage, the effective barrier height coincides with the Schottky barrier Φ_B,eff_(*V*_FB_) = Φ_Bn_. In detail, the *I*_d_ vs 1/*T* data at a given *V*_gs_ in the range −60 to 60 V are plotted in Figure 3c and linearly fitted to calculate the effective barrier Φ_B,eff_ at each gate bias
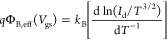
4

Φ_B,eff_ becomes smaller as the channel conduction band bends downward toward the source Fermi level. The plot Φ_B,eff_(*V*_gs_), shown in Figure 3d, is characterized by three different zones, which are consistent with the three marked areas in Figure 3b. A kink can also be observed here at *V*_gs_ ≈ – 20 *V* that corresponds to the flat-band condition, while the almost saturated profile at high voltages indicates that TT becomes the dominant mechanism. The Schottky barrier height qΦ_Bn_ = 0.091 eV is consistent with the estimated position of the trap centers that can cause Fermi level pinning.

Considering the electron affinity of ReS_2_ (∼4.30 eV)^37^ and the Cr work function (∼4.5 eV), the ideal Cr–Au/ReS_2_ Schottky barrier given by the difference between the electron affinity and the work function should be 0.20 eV.^38^ The extracted smaller value suggests that the Cr Fermi level is actually pinned near the ReS_2_ conduction band.^39^ The Fermi level pinning can be attributed to defect-induced gap states, which are also a source of free carriers.^40^ Atomic vacancies can, in fact, induce interface states that modify the contact properties.^39,41^

From the fit of the linear part of the *q*Φ_B,eff_(*V*_gs_) curve below *V*_fb_, we extract the body factor γ =  = 0.00161 ± 0.00004 and consequently estimate the capacitance of the localized states at the interface between the material and the oxide *C*_it_ ∼ (7.1 ± 0.2) × 10^–6^ F/cm^2^. Such a capacitance is related to the trap density *D*_it_ = *C*_it_/e^2^ ∼ (4.5 ± 0.2) × 10^13^ eV^–1^ cm^–2^.^42^

Figure 3e shows the field-effect mobility as a function of temperature, estimated from the linear part of the transfer curves for *V*_gs_ > *V*_th_. Mobility usually follows a trend in which it reaches a peak at a certain temperature. Prior to this critical temperature, the mobility is generally affected by scattering from charged impurities, and thereafter it decreases due to electron–phonon scattering.^43^ Some literature reports critical temperatures of *T* ∼ 120 K^18^ and *T* ∼ 100 K,^24^ and the two branches—the charge impurities and the phonon-scattering-dominated branches—are clearly visible. Here, the mobility increases with increasing temperature up to 270 K because of the ionization scattering but shows only a little variation in the 270–350 K range.^32^ The maximum mobility value is μ ∼ 3 cm^2^ V^–1^ s^–1^. This suggests that the device mobility is influenced not only by phonon scattering, which should dominate in the temperature range investigated, but also by charge traps.^19^ This requires further investigations into the scattering mechanisms in ReS_2_.

The behavior of the subthreshold swing as a function of temperature is reported in the inset of Figure 3e. It increases with temperature from 180 to 330 K. The lowering temperature causes an increase in both the *I*_on_/*I*_off_ ratio and *V*_th_ extracted from transfer curves on a linear scale. In detail, the *I*_on_/*I*_off_ ratio goes from about 300 at 350 K to 10^4^ at 180 K. The threshold voltage, reported in Figure 3f, ranges from about −15 at a high temperature to −12 at 180 K. It is identified by the *x*-axis intercept of the straight-line fitting of the transfer curve on a linear scale. The charge concentration values per unit area at *V*_gs_ = 60 V, computed according to the parallel-plate capacitor model: , where  and Δ*V* = *V*_gs_ – *V*_th_,^43^ range from 3.5 × 10^12^ to 5.5 × 10^12^ cm^–2^ as the temperature increases, which is consistent with the conductance behavior shown in Figure 3a.^24,29^

As already pointed out, the good electronic properties of ReS_2_ make it promising for photodetectors, as the direct band gap results in a high photogeneration rate and a high light absorption coefficient.

**Figure 4:**
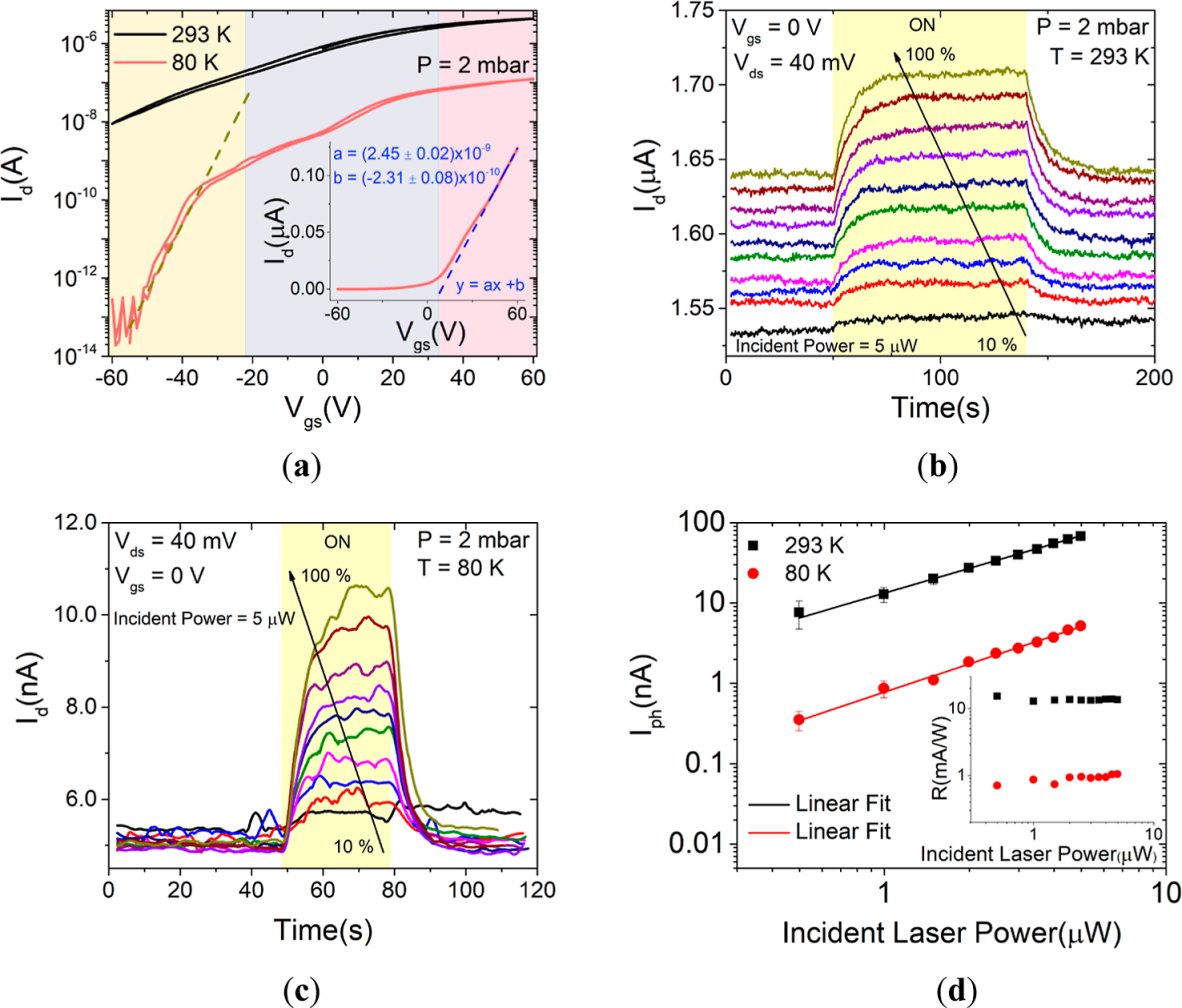
At 2 mbar: (a) Transfer characteristics on a semilog scale at both 80 and 293 K. (b) Drain current vs. time under 90 s laser pulses of increasing intensities at 293 K. (c) Drain current vs. time under 30 s laser pulses of increasing intensities at *T* = 80 K. (d) Photocurrent vs. incident laser power data at *T* = 293 K (black) and *T* = 80 K (red). A linear behavior occurs in both cases. Responsivity as a function of the incident laser power is reported in the inset.

First of all, we concentrate on the photoresponse at room temperature and low temperature. The transfer curves in the dark at 290 K (black) and 80 K (red) are shown in Figure 4a. It is confirmed that cooling the sample causes a decrease in the conductivity and suppresses the off current, giving an enhanced *I*_on_/*I*_off_ ratio. On the semilog scale, the three current mechanisms mentioned before, namely the thermionic-dominated region, the tunneling-dominated region, and the mixed transition region, are quite evident due to the change in slope. At the temperature of 80 K, using the linear fitting of the upper part of the transfer curve on a linear scale and the lower part of the transfer on a semilog scale, we obtained a mobility μ = 0.11 cm^2^ V^–1^ s^–1^ and a SS = 5.8 V/decade. Both values are lower than those at higher temperatures.

Figure 4b,c report the switching behavior of the device upon illumination by a supercontinuum white laser light of varying incident power from 0.5 to 5 μW and a spot of 1 mm in diameter. Hence, we studied the room- and low-temperature (80 K) photoresponses of the devices by monitoring the changes in the transient characteristics under light pulses of increasing intensities, for 90 and 30 s, respectively, while maintaining a constant *V*_gs_ = 0 V. At 290 K, the device shows a stable and repeatable response to repeated laser cycles. Figure 4b shows one pulse at each intensity. As soon as the laser is turned on, the current increases rapidly, until it almost saturates. As shown in Figure 4d, the photocurrent, defined as *I*_ph_ = *I*_laser_ – *I*_dark_, follows a linear trend with the laser power. The photocurrent magnitude is one order lower at 80 K but still shows a linear dependence on the laser power, as reported in Figure 4d. Linearity suggests that the photoconducting effect, namely the generation/recombination of photocarriers, is the main mechanism at both the temperatures investigated.^44^

Literature works generally report a power-law dependence of the photocurrent on the incident light power, *I*_ph_ ∝ *P*^γ^, with exponent γ < 1 at both room temperature and below, revealing a sublinear relation, even at incident power lower than the one reported herein.^19,45^ Several processes can lead to nonlinear power dependence, like photogating effect, which is very common in 2D layered materials,^46^ thermoelectric effects, trapping, etc. This behavior, which is typically observed in a wide variety of disordered semiconductors, can be attributed to the presence of a large energy distribution of recombination states in the gap.^45^ States that act as recombination centers are those (called ground states) located between the quasi-Fermi levels once the light excitation is on. The quasi Fermi levels, indeed, can be taken as an approximation of the demarcation lines between the shallow traps and the ground states. If there is a continuous distribution of states, the number of recombination centers rises when the light excitation is enhanced, for example, by increasing the power, because the two steady-state quasi Fermi levels move apart toward their respective band edges when the carrier concentration of excess electrons and holes increases. This reduces the carrier lifetime, making the photocurrent increase sublinearly with the laser power.^47^ The lack of this sublinear behavior suggests that the energy distribution of gap states in our sample is not so large and can presumably be described by one or a few energy levels.

Further, we calculated the rise and decay times by fitting the rise and decay trends with single exponentials, resulting in rise and decay times almost independent of the laser power. They are of around 5 s at the temperature of 80 K, and about 9 and 7 s, respectively, at 290 K. Rise and decay times at room temperature are shorter than previously reported values by other authors.^19,20^ Moreover, we note that at room temperature the repeated pulses lead to a slight current increase, probably due to the thermal effect. Conversely, at 80 K, the dark current returns to the initial state after each laser pulse with a shorter decay time.

We also evaluated the photoresponsivity as an important figure of merit. It is defined as *R* = *I*_ph_/*P*_inc_, where *P*_inc_ is the effective incident power that considers the area of the laser beam and the area of the flake. The inset of Figure 4d shows that the photoresponsivity is almost constant for all of the power intensities, consistent with the linear behavior of the photocurrent. It is around 1 mA/W at 80 K, and it is higher at room temperature (∼13 mA/W).

**Figure 5:**
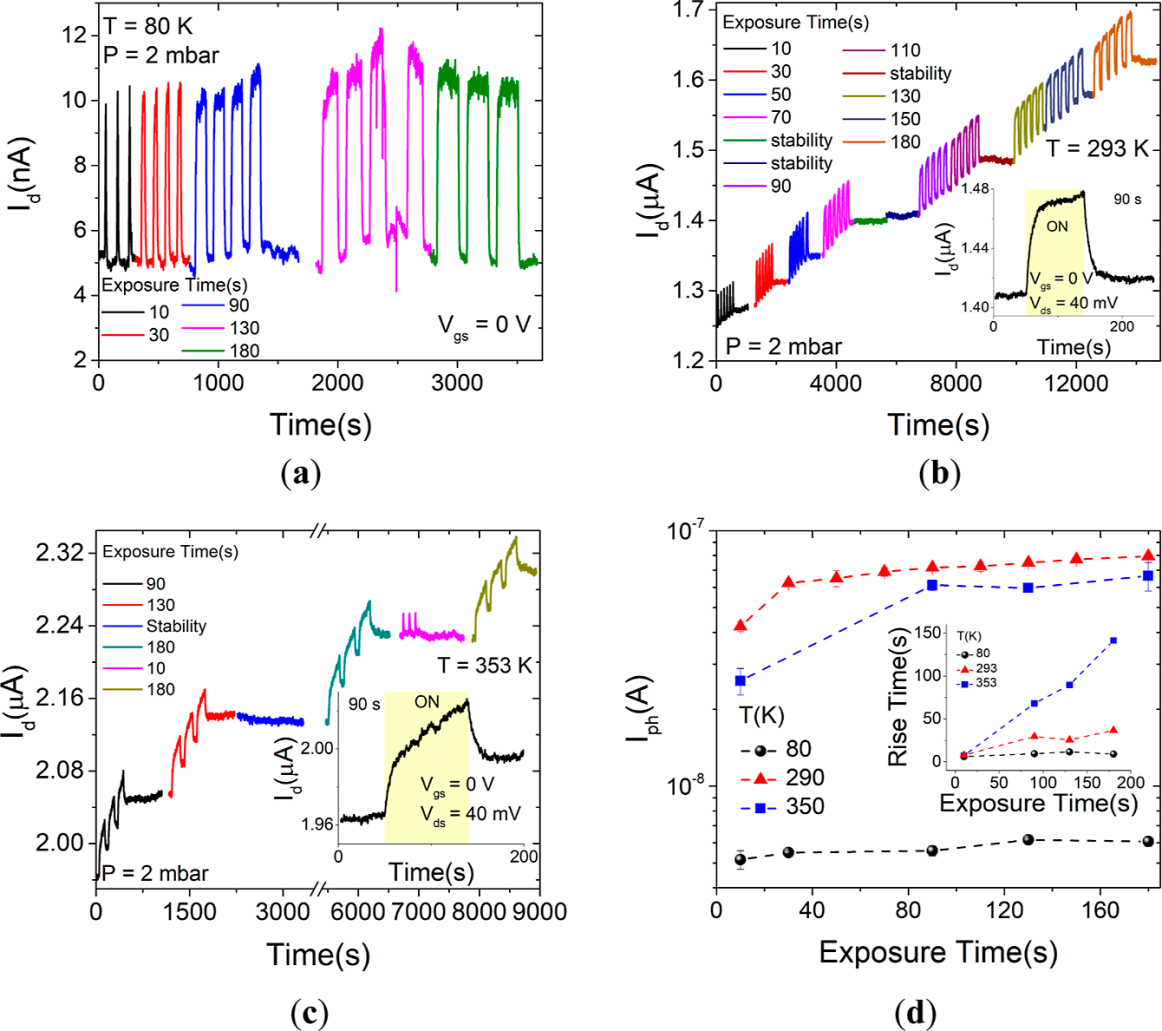
Drain current vs. time for several exposure times under the laser (a) at 80, (b) at 293 K (the inset shows a zoom-in view of the shape of the drain current under 90 s laser pulses), and (c) at 353 K (the inset shows a zoom-in view of the shape of the drain current under 90 s laser pulses). (d) Comparison of the photocurrent vs. the exposure time under the laser at 80, 293, and 353 K. The rise times as a function of the exposure times are reported for the three temperatures in the inset. Dashed lines are for eye guidance only. All measurements are at 2 mbar pressure.

We also analyzed the time-resolved photoresponse behavior of the device for increasing exposure times at different temperatures. Figure 5a–c show the response of the device when repeatedly illuminated by laser pulses of 104.5 mW of increasing duration at the temperatures of 80, 293, and 353 K, respectively. It can be noted that the maximum photocurrent is almost independent of the light pulse duration at low temperatures, while it slightly increases with it at higher temperatures. Most importantly, at room and higher temperatures, it is observed that performing laser pulses dramatically affects the background (dark) current, causing a steady overall increase of the current. Remarkably, the background current remains constant at the given level when the laser is kept off but undergoes a steady increase when the laser pulses are repeated. The rate of background current increase is higher at 353 K, indicating a temperature-related effect. Insets of Figure 5b,c also show that the background current increase drives the overall behavior of the photoresponse, indicating that the rise and decay times of the photocurrent substantially increase with the temperature, making the system not able to relax after repeated light pulses. As shown in the inset of Figure 5d, the rise times are actually longer at 353 K and are more dependent on the irradiation time. A temperature-dependent photoconductivity has been reported for other 2D materials, such as MoS_2_, and was attributed to the photobolometric effect and light-induced desorption of adsorbates.^48^ The photobolometric effect is related to the direct heating of the material by the incident radiation, which leads to a change in the physical parameters of the device and to a slower response.^49^ The light-induced desorption of adsorbates, facilitated at high temperature and low pressure, enhances the n-doping of ReS_2_, resulting in an increase in the current. In fact, Figure 5d shows an overall increasing trend of the photocurrent, going from 80 to 350 K. The observation of the highest photocurrent at 293 K rather than at 353 K is justified by the extracted mobility, which is higher at room temperature than at 350 K (Figure 3f), as the magnitude of the photocurrent is related to the transport properties. Moreover, at high temperatures, the enhancement of nonradiative processes makes the photocurrent be lower.^47^

## Conclusions

We investigated the electrical transport and the photoresponse in back-gate rhenium disulfide field-effect transistors over a wide temperature range. Current–voltage characterization as a function of temperature confirmed that the channel conductivity follows an activation law with activation energy around 100 meV. A Schottky barrier with a height comparable to the activation energy is formed at the contacts as an effect of Fermi level pinning close to the ReS_2_ conduction band. The device showed a strong photoresponse with photocurrent that increases with the temperature and depends linearly on the incident light power. The rise/decay times increase with increasing temperature. The time-resolved photocurrent can be ascribed to the photobolometric effect and light-induced desorption of adsorbates facilitated by the high temperature and low pressure.

## Materials and Methods

The device employed in the study was measured in a Janis ST-500 Probe Station (Lake Shore Cryotronics, Inc.), whose sample holder, which is in direct electrical contact with the Ag-pasted n-Si substrate, is used to apply the back-gate voltage, while two nanoprobes are connected to the source/drain metallic leads. The measurements were performed by the source measurement units of a Keithley 4200 SCS semiconductor characterization system. For the transistor characterization, the source was grounded, while drain and gate voltages were either swept or stepped according to the current–voltage (*IV*) test performed, and the drain and gate currents were monitored. In detail, transfer characteristics were obtained at a fixed drain voltage while forward and reverse sweeping the gate voltage. Similarly, output characteristics were obtained by sweeping the drain voltage, while the gate bias was varied in steps of 20 V.

The electrical measurements were carried out at a controlled pressure of 2 mbar. A supercontinuum white laser source with a maximum power of 110 mW and a wavelength in the 450–2400 nm range was used to investigate the photoresponse of the device.

Finally, Raman spectrometry was used to extract the vibrational properties of the investigated device. A laser with a wavelength of 532 nm, a spot size of 10 μm in diameter, and a power of 5 mW was employed as the excitation source. The spectra were acquired in the range 50–530 cm^–1^, while the 100–250 cm^–1^ spectrum was highlighted, with 1 s exposure time.
